# Literature Mining and Ontology based Analysis of Host-*Brucella* Gene–Gene Interaction Network

**DOI:** 10.3389/fmicb.2015.01386

**Published:** 2015-12-09

**Authors:** İlknur Karadeniz, Junguk Hur, Yongqun He, Arzucan Özgür

**Affiliations:** ^1^Department of Computer Engineering, Boğaziçi UniversityIstanbul, Turkey; ^2^Department of Basic Sciences, School of Medicine and Health Sciences, University of North Dakota, Grand ForksND, USA; ^3^Unit for Laboratory Animal Medicine, Department of Microbiology and Immunology, University of Michigan, Ann ArborMI, USA; ^4^Department of Computational Medicine and Bioinformatics, University of Michigan Medical School, Ann ArborMI, USA; ^5^Comprehensive Cancer Center, University of Michigan Health System, Ann ArborMI, USA

**Keywords:** host–pathogen interaction extraction, *Brucella*, text mining, host and pathogen gene name recognition, SciMiner, support vector machines (SVM), Interaction Network Ontology (INO)

## Abstract

*Brucella* is an intracellular bacterium that causes chronic brucellosis in humans and various mammals. The identification of host-*Brucella* interaction is crucial to understand host immunity against *Brucella* infection and *Brucella* pathogenesis against host immune responses. Most of the information about the inter-species interactions between host and *Brucella* genes is only available in the text of the scientific publications. Many text-mining systems for extracting gene and protein interactions have been proposed. However, only a few of them have been designed by considering the peculiarities of host–pathogen interactions. In this paper, we used a text mining approach for extracting host-*Brucella* gene–gene interactions from the abstracts of articles in PubMed. The gene–gene interactions here represent the interactions between genes and/or gene products (e.g., proteins). The SciMiner tool, originally designed for detecting mammalian gene/protein names in text, was extended to identify host and *Brucella* gene/protein names in the abstracts. Next, sentence-level and abstract-level co-occurrence based approaches, as well as sentence-level machine learning based methods, originally designed for extracting intra-species gene interactions, were utilized to extract the interactions among the identified host and *Brucella* genes. The extracted interactions were manually evaluated. A total of 46 host-*Brucella* gene interactions were identified and represented as an interaction network. Twenty four of these interactions were identified from sentence-level processing. Twenty two additional interactions were identified when abstract-level processing was performed. The Interaction Network Ontology (INO) was used to represent the identified interaction types at a hierarchical ontology structure. Ontological modeling of specific gene–gene interactions demonstrates that host–pathogen gene–gene interactions occur at experimental conditions which can be ontologically represented. Our results show that the introduced literature mining and ontology-based modeling approach are effective in retrieving and analyzing host–pathogen gene–gene interaction networks.

## Introduction

*Brucella* is a Gram-negative intracellular bacterium that causes zoonotic brucellosis in humans and various animals. Brucellosis is one of the most common zoonotic diseases worldwide, causing approximately half a million new human brucellosis each year. There are 10 species of *Brucella* based on the preferential host specificity: *Brucella melitensis* (goats), *B. abortus* (cattle), *B. suis* (swine), *B. canis (*dogs), *B. ovis* (sheep), *B. neotomae* (desert mice), *B. cetaceae* (cetacean), *B. pinnipediae* (seal), *B. microti* (voles), and *B. inopinata* (unknown) (O’Callaghan and Whatmore, 2011). Among them, *B. melitensis*, *B. abortus*, *B. suis*, and *B. canis* are pathogenic to human. The other *Brucella* species are non-pathogenic to humans.

The genome sequences of all *Brucella* species are strikingly similar with nearly identical genetic content and gene organization (Halling et al., 2005). Humans can be infected with *Brucella* by contact with infected animals, by inhalation of an aerosol, or by ingestion of contaminated animal products (e.g., infected milk and meat). Upon entry into animals, the bacteria invade the blood stream and lymphatics where they multiply inside phagocytic cells and eventually cause septicemia. Symptoms include undulant fever, abortion, asthenia, endocarditis and encephalitis. In spite of a long documented history (Corbel, 1997), the treatment of human brucellosis remains difficult and requires antibiotics that penetrate macrophages and can act in an acidic intracellular environment. While currently used live attenuated *Brucella* animal vaccines (e.g., RB51, strain 19, and Rev. 1) have the ability to protect animals, they are still pathogenic to humans. No safe and effective *Brucella* vaccine is available for human use. To develop safe and effective preventive and therapeutic measures against *Brucella* infections, it is critical to understand the host-*Brucella* mechanisms that lead to *Brucella* pathogenesis and host immunity against *Brucella* infection. Although extensive studies have been undertaken, the systematic understanding of the host-*Brucella* interactions is still missing.

**FIGURE 1:**
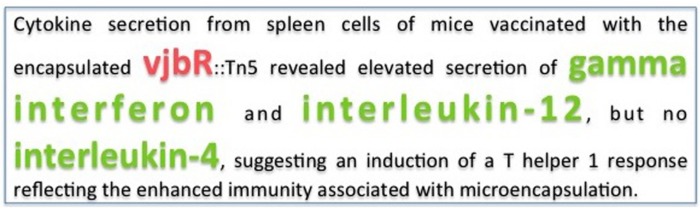
**Sample host–pathogen interaction describing sentence (Arenas-Gamboa et al., 2008).** The pathogen gene is shown in red and the host genes are shown in green.

Currently, there is very limited information regarding host-*Brucella* interactions in the host–pathogen interaction databases such as PHIDIAS (Xiang et al., 2007), PHISTO (Tekir et al., 2013), and HPIDB (Kumar and Nanduri, 2010). Most of the relevant information is only available in a textual format in the published scientific articles. In this study, our goal is to utilize text mining methods to extract host-*Brucella* gene interactions from the biomedical literature. In order to extract host–pathogen gene interactions, first the pathogen and host gene names should be identified in text, then the interactions among the host and pathogen genes should be detected. For example, the sentence shown in **Figure 1** (Arenas-Gamboa et al., 2008) contains three host genes *(gamma interferon, interleukin-12*, and *interleukin-4*) and one pathogen gene (*vjbR)*. This sentence states that there are two pathogen–host gene interactions: *(gamma interferon, vjbR)* and *(interleukin-12, vjbR).* On the other hand, there is no an interaction between the host gene *interleukin-4* and pathogen gene *vjbR.*

Different methods have been proposed for literature mining of gene–gene interactions. One of the simplest and widely used methods is based on the co-occurrence statistics of the proteins in text (Jelier et al., 2005). Another common approach is matching pre-specified patterns and rules over the sequences of words and/or their parts of speech in the sentences (Ono et al., 2001; Blaschke and Valencia, 2002). More recently, machine learning methods that integrate the linguistic, syntactic, and/or semantic analysis of the sentences as kernel functions have been proposed and shown to achieve state-of-the-art results for gene/protein interaction extraction from text (Giuliano et al., 2006; Erkan et al., 2007; Airola et al., 2008; Tikk et al., 2010). Similarly to previous literature mining studies, in this paper we used the commonly applied GENETAG-style named entity annotation (Tanabe et al., 2005). In other words, a gene interaction can involve genes or gene products such as proteins.

A number of rule-based and machine learning based methods have been proposed for identifying gene/protein mentions in text (Fukuda et al., 1998; McDonald and Pereira, 2005; Tsai et al., 2006; Hsu et al., 2008). In our previous studies, we developed dictionary- and rule-based named entity recognition tools, SciMiner (Hur et al., 2009) and Vaccine Ontology (VO)-SciMiner (Hur et al., 2011), which are designed to identify genes/proteins and Vaccine Ontology (VO) terms in the biomedical literature. Conventional Medical Subject Headings (MeSH) terminology has been frequently used for literature mining, such as GenoMesh studies (Xiang et al., 2013). The usage of ontologies enhances the chances of retrieving gene–gene interactions. For example, in our recent studies we have shown that the VO facilitates the retrieval of vaccine-associated IFN-gamma interaction network (Özgür et al., 2011), fever-related network (Hur et al., 2012), and *Brucella* vaccine interaction network (Hur et al., 2012). Recently, we have developed an Interaction Network Ontology (INO) which is used to classify the interaction keywords such as up-regulation, inhibition, association, and binding in an ontology structure (Hur et al., 2015). The classified interaction hierarchy makes us not only retrieve gene–gene interactions, but also the types of gene–gene interactions (Hur et al., 2015). We hypothesize that such a strategy can also be used in host–pathogen gene–gene interaction literature retrieval.

Currently, the research in host–pathogen interactions literature mining mostly focuses on the retrieval of host gene–gene interaction under a particular pathogen infection (e.g., influenza) or pathogen gene–gene interactions [e.g., our *Brucella* vaccine interaction network analysis (Hur et al., 2012)]. There are only a few studies on the retrieval of both host and pathogen genes and the inter-species interactions among them [reviewed in (Durmus et al., 2015)]. Machine learning based methods were proposed for classifying abstracts of scientific articles as being relevant to host–pathogen interactions or not (Yin et al., 2010; Thieu et al., 2012). In addition, Thieu et al. (2012) proposed a rule-based approach that is based on the link-grammar representations of the sentences for extracting host–pathogen protein interactions from text.

In this study, we use kernel-based methods for extracting host–pathogen gene interactions, which have been shown to achieve promising results for extracting intra-species protein interactions (Erkan et al., 2007; Tikk et al., 2010). One main issue in host–pathogen interaction literature mining is the confusion of a gene being a host gene or pathogen gene, since many gene names are shared in both hosts and pathogens. This is one main research topic in our current study. We extended the SciMiner mammalian gene name identification tool to recognize and distinguish between host and *Brucella* genes. In addition, we used an INO-based method to model various gene–gene interactions under different experimental conditions. Our results show that our combinatory strategy is able to successfully retrieve and analyze host–pathogen gene–gene interaction networks.

## Materials and Methods

The main focus of this study is to identify the interactions between host and *Brucella* genes. Many eukaryotic organisms act as the host of *Brucella* infections, including human, cattle, goat, sheep, pig, etc. As a laboratory animal model, mice can also be infected with *Brucella*. Our literature mining study covers these different host species. Meanwhile, there are 10 different *Brucella* species.

**FIGURE 2:**
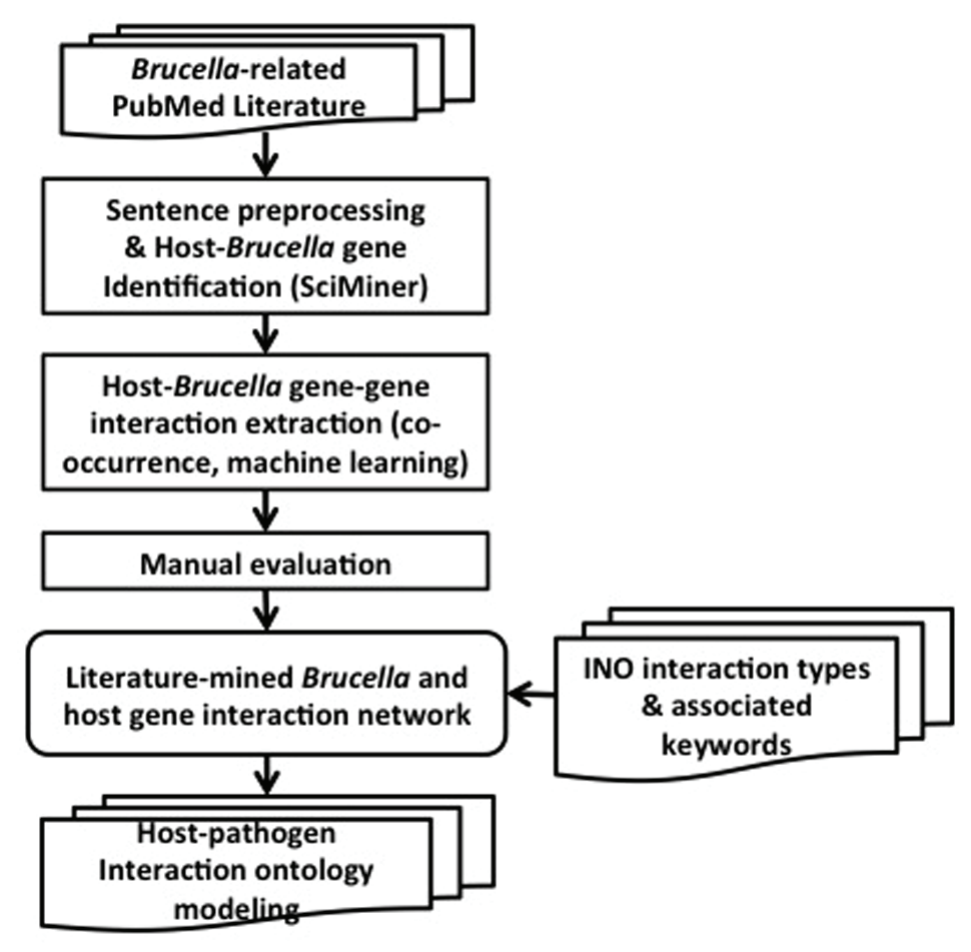
**Project design pipeline and workflow**.

The overall design and workflow of our approach is shown in **Figure 2**. All PubMed papers are used as our data sources. They are filtered based on their relevance to *Brucella*. The selected abstracts are processed by splitting into sentences and identifying the host and *Brucella* gene name mentions using SciMiner. Next, co-occurrence and machine learning based methods are used to extract the interactions among the host and *Brucella* genes. A literature-mined and manually verified host-*Brucella* gene–gene interaction network is created. Finally, ontology based modeling of host–pathogen gene–gene interactions is performed by utilizing the INO. The details of the methods are presented in the following subsections.

### Data Set Collection

The 2015 MEDLINE^®^/PubMed^®^ Baseline Distribution database consisting of 23,343,329 records was downloaded from the US National Library of Medicine and processed using our established literature mining pipeline. Briefly, the title, abstract, and MeSH terms of each record were parsed out from the downloaded XML files. The collected abstracts were split into sentence level using Java’s LBJ2.nlp.SentenceSplitter module. Then, enhanced version of our named entity recognition tools, SciMiner (Hur et al., 2009) and VO-SciMiner (Hur et al., 2011), were used to identify host genes and pathogen genes, and the results were populated into a local MySQL database. To define the *Brucella-*specific context, we used a PubMed query, “*Brucella* OR Brucellosis,” which resulted in a list of 16,699 PubMed IDs as of 2/1/2015.

### Identifying Gene Names

To identify the mentioned host genes and *Brucella* genes in the abstracts of articles, we used our in-house named entity recognizers, SciMiner^1^ (Hur et al., 2009) and VO-SciMiner^2^ (Hur et al., 2011). SciMiner and VO-SciMiner are both dictionary- and rule-based literature mining tools. SciMiner focuses on identification of mammalian genes, reported in terms of the official human genes based on the HUGO Gene Nomenclature Committee (HGNC) database^3^, while VO-SciMiner identifies VO terms and *Brucella* genes.

In the present study, to improve identification accuracy of host and pathogen genes, we enhanced the mining rules in both SciMiner and VO-SciMiner. First, the enhanced version of SciMiner uses a stringent case-match of gene symbols. In the original version of SciMiner, which included dictionary of only human genes names and symbols, a relaxed matching of symbols was employed to maximize the gene identification (high recall). This relaxed case matching resulted in misidentifications such as *recA*, recombinase A gene, being identified as the human RAD51 recombinase (RAD51), whose aliases include RECA. Since the majority of the *Brucella* gene symbols start with a lower-case character and usually end with an upper-case or numeric character, SciMiner excluded symbols with this pattern. In case of the genes identified by both SciMiner as a host gene and VO-SciMiner as a pathogen gene, the priority is given to the VO-SciMiner identification considering the current context of *Brucella*-related literature.

### Mapping Genes to Pathogen and Host Species

In order to further improve the overall accuracy of host gene identification, we used potential host species-related MeSH terms, including ‘humans,’ ‘rats,’ ‘mice,’ ‘cattle,’ ‘guinea pigs,’ ‘swine,’ ‘goats,’ and ‘sheep’ to filter the genes identified by SciMiner. Only the host genes identified from PubMed documents whose MeSH terms included at least one of these selected terms were included for further analysis.

### Gene–gene Interaction Extraction

In this study, co-occurrence based and machine-learning based approaches are used for extracting host–pathogen gene–gene interactions. Both sentence-level and abstract-level co-occurrence approaches, as well as a machine learning-based approach are investigated for this task. These approaches are described in the following subsections.

#### Co-occurrence Based Host–pathogen Interaction Extraction

We used two different contexts to extract the interactions based on the co-occurrences of the host and pathogen genes: sentence-based context and abstract-based context. In the sentence-based co-occurrence approach, if one pathogen and one host gene occur in the same sentence, an interaction pair is extracted consisting of the corresponding pathogen and host genes. For example, in the sentence shown in **Figure 1** (Arenas-Gamboa et al., 2008), the SciMiner tool identifies two host genes *(interleukin-12* and *interleukin-4*) and one pathogen gene (*vjbR)*. The sentence-level co-occurrence approach extracts the interactions *(interleukin-12, vjbR)* and *(interleukin-4, vjbR)* from the sample sentence, where *(interleukin-12, vjbR)* is a true interaction and *(interleukin-4, vjbR)* is an incorrectly extracted interaction. In the sample sentence, *gamma interferon* is also a host gene. However, since this gene is not detected by SciMiner, it is not considered in the interaction extraction step. In the abstract-based co-occurrence approach, an abstract is taken into consideration as the context window instead of a single sentence. In other words, all pairs of host and pathogen genes that occur in the same abstract are extracted as interacting pairs regardless of the sentence boundaries.

#### Machine Learning Based Host–pathogen Interaction Extraction

We utilized a machine learning based approach to classify whether a host and pathogen gene pair occurring in the same sentence is described as interacting in the sentence or not. We used support vector machines (SVM) [specifically the SVM*^light^* package (Joachims, 1999)] as our classification algorithm with the cosine and edit kernels introduced in (Erkan et al., 2007). These kernels make use of the dependency parse trees of the sentences that represent the syntactic and semantic relations among the words. We used the Stanford Parser (de Marneffe et al., 2006) to obtain the dependency parse trees of the sentences in our *Brucella* specific data set. We only processed sentences for which SciMiner identified at least one host and one pathogen gene. The cosine and edit kernels are defined over the path between the host gene and pathogen gene in the dependency parse tree of the corresponding sentence.

**FIGURE 3:**
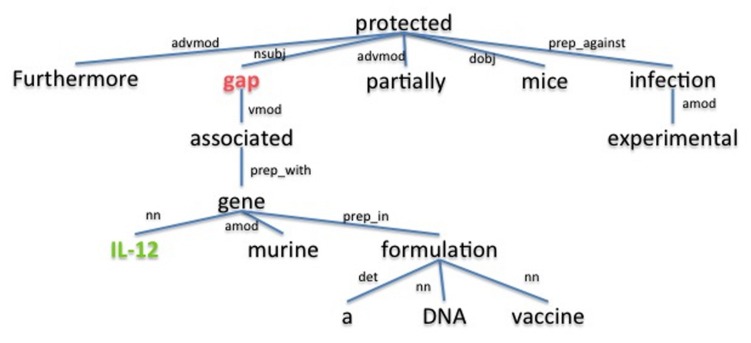
**The dependency parse tree of a sample sentence.** The tree is generated for the sentence “Furthermore, gap associated with murine *IL-12* gene in a DNA vaccine formulation partially protected mice against experimental infection.” from the abstract of (Rosinha et al., 2002). Host and pathogen genes identified by SciMiner are shown in green and red, respectively. The Stanford parser was used to generate the parse tree. advmod, adverb modifier; amod, adjectival modifier; det, determiner; dobj, direct object; nn, noun compound modifier; nsubj, nominal subject; prep_against, preposition against; prep_in, preposition in; prep_with, preposition with; vmod, reduced non-finite verbal modifier.

The underlying assumption is that the dependency path between a host and a pathogen gene is a good description for the relation between them. For example, the dependency parse tree obtained using the Stanford parser (de Marneffe et al., 2006) for the sample sentence “Furthermore, *gap* associated with murine *IL-12* gene in a DNA vaccine formulation partially protected mice against experimental infection.” (Rosinha et al., 2002), is shown in **Figure 3**. The dependency path between the host gene *IL-12* and the pathogen gene *gap*, which are described as interacting in the given sentence, is “nn gene prep_with associated vmod.” On this path we have the word *associated* as well as the dependency relation type *preposition with (prep_with)*, which provide clues for the interaction between *gap* and *IL-12.* Using the cosine similarity and edit distance kernel functions within SVM (Erkan et al., 2007), our program is able to infer whether or not these two genes interact with each other. Note that this sentence also includes the gene symbol “gap” which is a common English word. SciMiner has a confidence scoring system for each identified gene symbol in the text, based on weighted co-occurrences of the gene symbol and their descriptions (e.g., gene or protein names) in the same text. In this case, since the protein name of the gap gene “glyceraldehyde-3-phosphate dehydrogenase” is described in the paper abstract, the SciMiner scoring system was able to assign gap as a gene.

To the best of our knowledge, there are no publicly available manually labeled host–pathogen gene–gene interaction corpora. Therefore, we trained the SVM classifier with edit and cosine kernels by using corpora labeled for intra-species protein–protein interactions. Specifically, we used the Christina Brun (CB) corpus provided as a resource at the BioCreAtIve II challenge^4^ and the AIMED corpus (Bunescu et al., 2005), which is a standard corpus for evaluating intra-species protein–protein interactions. The learned cosine and edit kernel based SVM models are used to classify each sentence as an interaction-describing sentence (positive class) or not (negative class) for each host and pathogen gene pair identified by SciMiner in the corresponding sentence.

### Evaluation

The results obtained by the co-occurrence and machine learning based interaction classification methods (i.e., classifiers) are manually evaluated by using the number of TP (True Positives), FP (False Positives), TN (True Negatives), and FN (False Negatives), as well as the precision, recall, and *F*-score metrics.

True Positives is the number of host–pathogen interactions correctly classified as positive; FP (False Positives) is the number of negative host–pathogen interactions that are incorrectly classified as positive by the classifier; TN (True Negatives) is the number of host pathogen interactions classified correctly as negative (no interaction); and FN (False Negatives) is the number of positive host–pathogen interactions that are incorrectly classified as negative by the classifier.

Precision is the ratio of correctly identified positive host–pathogen interactions over all interactions classified as positive by the classifier [i.e., TP/(TP + FP)]. Recall is the ratio of correctly classified positive host–pathogen interactions over all positive host–pathogen interactions [i.e., TP/(TP + FN)]. *F*-score is the harmonic mean of these two measures [i.e., 2 . precision . recall/(precision + recall)].

### Ontology Modeling

The INO focuses on the ontological representations of hierarchical biological interaction types and networks (Hur et al., 2015). INO has been proven to enhance the literature mining of gene–gene interaction types (Hur et al., 2015). In this study, we applied INO to analyze different interaction types between host and *Brucella* at different experimental conditions. Furthermore, different conditions of host-*Brucella* interactions were represented and analyzed through ontology-based modeling.

## Results

### Identification of Host and *Brucella* Gene Names

Two of our in-house named entity recognizers, SciMiner and VO-SciMiner, were enhanced in our study to identify host and pathogen genes, respectively. First, SciMiner has been modified to use stringent case-match. In the context of *Brucella*, consisting of 16,699 PubMed abstracts, the enhanced versions of SciMiner and VO-SciMiner identified 47 unique pairs of potential host gene and *Brucella* gene interactions using the improved symbol-based identification method and confliction resolution between host and *Brucella* gene. Out of these 47 pairs, manual examination confirmed that 24 unique pairs were true interactions, indicating an overall accuracy of 51%.

### Identification of Host-*Brucella* Gene–gene Interactions

After identifying the host and *Brucella* gene names in sentences co-occurrence and machine learning based methods are used to classify each pair in a sentence as an interaction (positive class) or not (negative class). We performed manual evaluation for the classification decisions of the methods for each host-*Brucella* gene pair in each sentence. For the abstract-level co-occurrence approach, manual evaluation is performed for each host-*Brucella* gene pair in each abstract.

The results obtained are summarized in **Table 1**. Co-occurrence based methods classify all pairs of host–pathogen genes as positive, if they occur in the same sentence or abstract. Therefore, they obtain the maximum level of recall, i.e., 100%. Not all co-occurring gene pairs are true interaction pairs. For example, in the sample sentence shown in **Figure 1**, there is no an interaction between the pathogen gene *vjbR* and the host gene *interleukin-4.* However, the co-occurrence methods incorrectly classified this pair as interacting, since these genes occur in the same sentence. This leads to drop in precision.

**Table 1:** Co-occurrence and machine learning based host-*Brucella* gene–gene interaction results.

	TP	TN	FP	FN	Precision	Recall	*F*-score
Co-occurrence (sentence-based)	29	0	25	0	0.54	1.0	0.70
Co-occurrence (abstract-based)	55	0	61	0	0.47	1.0	0.64
Support vector machines (SVM; edit kernel)	15	12	12	14	0.56	0.52	0.54
SVM (cosine kernel)	12	19	5	17	0.71	0.41	0.52


*TP, True Positive; TN, True Negative; FP, False Positive; FN, False Negative.*

Support vector machines with edit and cosine kernel obtained a higher precision compared to the co-occurrence based approach. The precision obtained by the cosine kernel (71%) was significantly higher than the precision values of the co-occurrence and edit kernel approaches. Edit kernel, on the other hand, obtained more balanced precision and recall levels compared to the other methods.

Both edit kernel and cosine kernel operate on sentence-level. Therefore, they are not able to identify interactions whose descriptions cross sentence boundaries. The significantly higher number of true positive interactions retrieved by the abstract-level co-occurrence approach indicates the importance of the use of abstracts (or scopes wider than sentences) as context.

**FIGURE 4:**
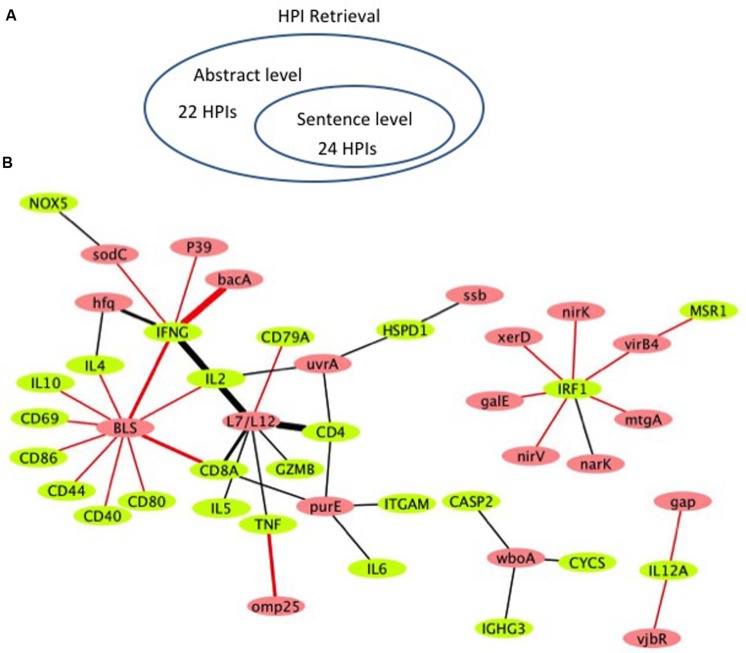
**Literature-mined host-*Brucella* gene–gene interaction results. (A)** Venn diagram showing the number of unique host-*Brucella* interaction gene pairs retrieved and manually verified from sentence-level and abstract-level processing. **(B)** The literature-mined and manually verified host-*Brucella* gene–gene interaction network. Host genes are shown in green and *Brucella* genes are shown in red. Red edges correspond to interactions retrieved from sentence-level processing. Black edges correspond to interactions retrieved from abstract level processing. The more sentences/abstracts describe an interaction between gene pairs the thicker the edge connecting them.

**Figure 4** shows the literature mined and manually verified unique host-*Brucella* gene–gene interactions. A total of 46 unique interaction pairs are retrieved. 24 of these were identified using sentence-level processing. Abstract-level analysis enabled the retrieval of 22 additional unique interaction pairs (**Figure 4A**). The identified host-*Brucella* gene–gene interactions are represented as a network, which consists of 20 *Brucella* genes and 25 host genes (**Figure 4B**). The interactions between host and *Brucella* gene pairs are represented as edges. The edges are weighed based on the number of sentences/abstracts that state the corresponding interaction. BLS and L7/L12 are the most connected *Brucella* genes, whereas IFNG and IRF1 are the most connected host genes.

### Ontology Modeling of Host-*Brucella* Gene–gene Interactions

**FIGURE 5:**
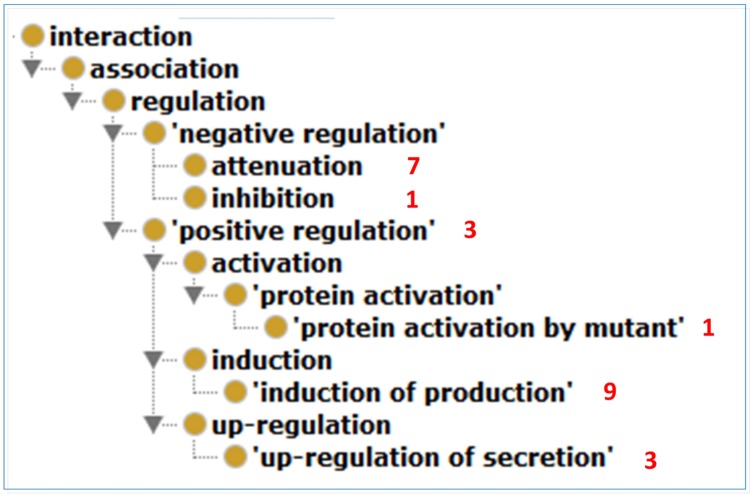
**The ontology hierarchy of literature mined INO interaction types.** In total, six different INO interaction types were identified from this literature mining study. The number of interactions of a specific type is shown in red next to the interaction type. The ‘induction of production’ type is the most common type identified.

We used INO to analyze the types of interactions between the extracted host and *Brucella* genes. The results of this analysis are shown in **Figure 5**. In total, six different INO interaction types, all of which are sub-types of regulation, are identified from this literature mining study. The ‘induction of production’ type is the most common type identified. For instance, the sentence “The P39 and the bacterioferrin (BFR) antigens of *B. melitensis* 16M were previously identified as T dominant antigens able to induce both delayed-type hypersensitivity in sensitized guinea pigs and *in vitro* gamma interferon (IFN-gamma) production by peripheral blood mononuclear cells from infected cattle” (Al-Mariri et al., 2001) is an example sentence that describes an interaction of type ‘induction of production’ between pathogen and host genes. The sentence states that *Brucella* gene P39 is able to induce *in vitro* host IFN-gamma production.

While **Figure 4** provides concrete summary of the host-*Brucella* gene–gene interaction network, it is typical that each gene–gene interaction occurs under specific experimental condition(s). Without a specific condition, any host–pathogen interaction will not happen. Ontology provides an ideal platform to model and represent these gene–gene interactions under specific conditions. Below we provide two examples to illustrate how ontology-based gene–gene interactions work. These two examples include one retrieved from sentence level literature mining and another from abstract level literature mining. The ontology modeling uses the framework of the INO (Hur et al., 2015), the Ontology for Biomedical Investigations (OBI; Brinkman et al., 2010), and the Brucellosis Ontology (IDOBRU; Lin et al., 2011, 2015).

**FIGURE 6:**
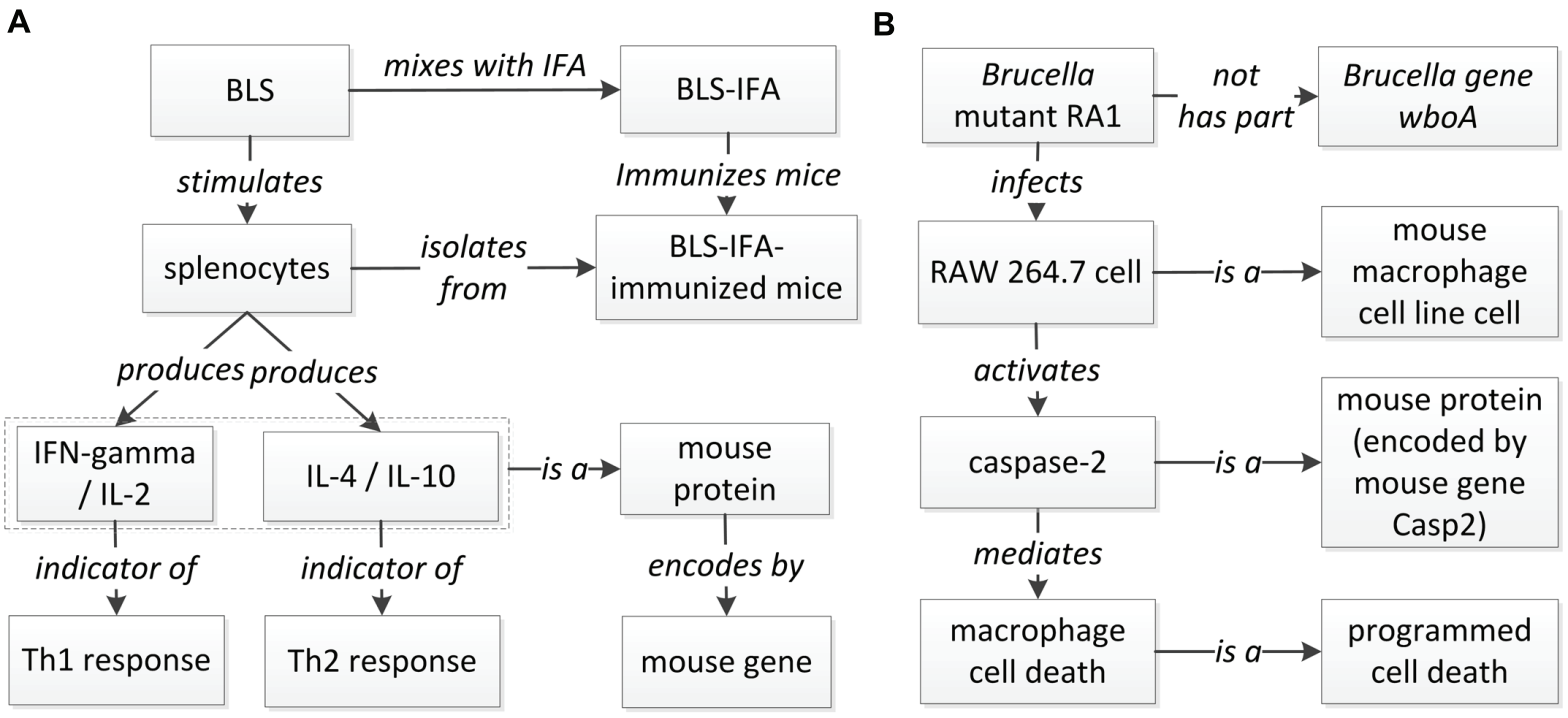
**Ontology modeling of literature-mined host-*Brucella* interaction types. (A)** Ontology modeling of the gene interaction from the sentence “In addition, after *in vitro* stimulation with rBLS, spleen cells from BLS-IFA-, BLS-Al-, or BLS-MPA-immunized mice proliferated and produced interleukin-2 (IL-2), gamma interferon (IFN-gamma), IL-10, and IL-4, suggesting the induction of a mixed Th1-Th2 response” (Velikovsky et al., 2003). **(B)** Ontology modeling of the Casp2-wboA gene interaction using the abstract content from the paper (Chen and He, 2009).

A host-*Brucella* gene–gene interaction based on literature mined sentence (Velikovsky et al., 2003) was modeled using ontology (**Figure 6A**). In this example, the mice were immunized with recombinant *Brucella* lumazine synthase (rBLS) administered with different adjuvants including incomplete Freund’s adjuvant (IFA), monophosphoryl lipid A (MPA), and aluminum hydroxide gel (Al). The splenocytes were isolated from immunized mice and then re-stimulated with rBLS. Different cytokines (IFN-gamma, IL-2, IL-4, and IL-10) were produced by the splenocytes, indicating a mix of Th1 and Th2 response. This model represents the detail of the interactions between *Brucella* BLS and mouse IFN-gamma, IL-2, IL-4, and IL-10. This example is classified as another ‘induction of production’ interaction type (**Figure 5**), i.e., recombinant BLS induces the production of different proteins in splenocytes isolated from immunized mice.

**Figure 6B** provides another example of ontology modeling of the interaction between *Brucella* gene *wboA* and mouse protein Caspase-2, encoded by mouse gene Casp2, using the abstract content from the paper (Chen and He, 2009). *Brucella* mutant RA1, a mutant of wild type, virulent *B. abortus* strain 2308, lacks the *Brucella* gene *wboA*. RA1-infected RAW 264.7 mouse macrophage cell line cells had activated Caspase-2, which mediated apoptotic and necrotic cell death of RAW 264.7 cells (Chen and He, 2009). This example represents how *Brucella* gene *wboA* interacts with mouse Caspase-2. This example demonstrates the interaction type of ‘protein activation by mutant’ (**Figure 5**), i.e., a mutant of a gene infects mouse macrophages and activates the production of a mouse protein Caspase-2.

## Discussion

Using *Brucella* as an example pathogen, this study utilized literature mining and ontology analysis approaches to examine the interactions between host genes/proteins and *Brucella* genes/proteins. Since genes encode for proteins, our host-*Brucella* gene–gene interactions also include protein–protein interactions. Our approach identified 46 pairs of host-*Brucella* gene–gene interactions from the literature, and the ontology modeling analysis identified different types of interactions and provided deeper insights on how the host and *Brucella* genes/proteins interact at different experimental conditions.

One challenge in host–pathogen interaction literature mining is the difficulty in differentiating host genes and pathogen genes. In the current version of SciMiner and VO-SciMiner we did not use any of the name (longer description)-based identification results in the analysis. This is due to our manual evaluation of the preliminary results suggesting it is far more difficult to distinguish between host and pathogen genes using longer description protein names as they are more redundant than gene symbols. For example, the protein name “Superoxide dismutase [Cu-Zn]” may represent a human/host gene name (SOD1 or SODC) or a *Brucella*/pathogen protein (SodC). In general, the gene names are more unique than the gene symbols; therefore, use of only short gene symbols resulted in decreased numbers of identified genes by the current versions of SciMiner and VO-SciMiner. We will examine these missed genes and further improve the sensitivity and accuracy of the gene name-based identification.

We investigated using co-occurrence and machine learning based methods for extracting host–pathogen gene–gene interactions. The co-occurrence based methods classify each pair of host and pathogen genes as interacting, if they occur in the same sentence/abstract. Therefore, they obtain high recall by retrieving all interacting pairs of genes. However, they also classify many gene pairs incorrectly as interacting, since not all co-occurring gene pairs are true interactions. This leads to drop in performance in terms of precision. The SVM classifiers with the dependency tree based edit and cosine kernels make use of the syntactic analysis of the sentences. These methods achieved higher precision compared to the co-occurrence based methods. To the best of our knowledge, there does not exist a large manually labeled host–pathogen gene–gene interaction data set. Therefore, the edit and cosine kernel based SVM classifiers were trained by using generic (intra-species) protein–protein interaction data sets. Training these classifiers with host–pathogen gene–gene interaction data might improve their performances. A drawback of most (if not all) currently available machine learning based interaction extraction methods is that they operate on sentence-level and therefore, are not able to identify interactions that cross sentence boundaries. As our sentence-level and abstract-level co-occurrence analysis revealed, many host-*Brucella* interactions span multiple sentences. These results suggest that developing text mining methods that operate on scopes wider than a sentence would be useful for extracting host–pathogen gene–gene interactions.

Our ontology modeling studies demonstrate its value in further identifying the nature and insights of host–pathogen gene–gene interactions. A simple gene–gene interaction may miss many details, especially in the setting of a host–pathogen interaction. A gene–(interaction type)-gene would provide more details since the interaction type could indicate how the two genes interact. The INO provides a way to classify hundreds of interaction keywords into logically defined interaction types under a hierarchical ontology setting (Hur et al., 2015). The usage of INO interaction types and its hierarchy allows us to detect the distribution of the interaction types from our literature mining study (**Figure 5**). INO-based modeling also provides a novel way to identify interaction types that are represented by multiple keywords in sentences (Özgür et al., 2015). Furthermore, ontology modeling of the mined sentences or abstracts provides a way to deeply identify the experimental setting where a host gene and a pathogen gene interact. Without such settings, detected host–pathogen interactions may not occur. Therefore, the ontology modeling is critical for our better detection and representation of the details of host–pathogen interaction mechanisms.

A promising future work is to use ontology modeling to identify possible types of patterns of how host and pathogen genes interact and apply such design patterns to guide our literature mining. For example, based on the ontology model of the ‘protein activation by mutant’ interaction type (**Figure 6B**), we may design a pattern-specific literature mining study. Specifically, a mutant represents a recombinant organism with the mutation of an internal gene. After a mutant is generated, a name is usually assigned to the mutant. As shown in **Figure 6B**, a pathogen mutant is often used in different experimental settings to infect a host and activate a host protein. Such a complex pattern is difficult to retrieve using current literature mining strategies. For instance, a sentence often describes the relation between a mutant (instead of a pathogen gene) and a host gene. Based on the ontology-modeled pattern, we can first design a literature mining approach to identify all mutants and their corresponding pathogen genes; and based on the mutant-gene interaction, we can then infer the gene–gene interaction. Specific experimental conditions (e.g., host cell types) can also be mined using the ontology modeling. Literature mined and experimentally verified results can further be ontologically represented in an ontology such as the Brucellosis Ontology (IDOBRU; Lin et al., 2011, 2015).

Compared to model pathogens such as *Escherichia coli* and *Salmonella*, *Brucella* is a less studied pathogen. However, the results obtained from this study provide the first example of opportunities and challenges in the literature mining of the host–pathogen gene–gene interactions.

## Conflict of Interest Statement

The authors declare that the research was conducted in the absence of any commercial or financial relationships that could be construed as a potential conflict of interest.

## Footnotes

1 http://jdrf.neurology.med.umich.edu/SciMiner/

2 http://www.violinet.org/vo-sciminer/

3 http://www.genenames.org/

4 http://biocreative.sourceforge.net/biocreative_2.html
